# Factors affecting commencement and cessation of smoking behaviour in Malaysian adults

**DOI:** 10.1186/1471-2458-12-207

**Published:** 2012-03-19

**Authors:** Wan Maria Nabillah Ghani, Ishak Abdul Razak, Yi Hsin Yang, Norain Abu Talib, Noriaki Ikeda, Tony Axell, Prakash C Gupta, Yujiro Handa, Norlida Abdullah, Rosnah Binti Zain

**Affiliations:** 1 Oral Cancer Research & Coordinating Centre, Faculty of Dentistry, University of Malaya, Kuala Lumpur, Malaysia; 2 Department of Dental Hygiene, College of Dental Medicine, Kaohsiung Medical University, Kaohsiung, Taiwan; 3 Oral Health Division, Ministry of Health, Kuala Lumpur, Malaysia; 4 Bureau of International Medical Cooperation, National Center for Global Health and Medicine, Tokyo, Japan; 5 Maxillofacial Unit, Hallands Hospital, Halmstad, Sweden; 6 Healis - Sekhsaria Institute For Public Health, Navi Mumbai, India; 7 Health Sciences University of Hokkaido, Tobetsu (Ishikari), Hokkaido, Japan

**Keywords:** Tobacco, Commencement, Cessation, Prevalence, Epidemiology

## Abstract

**Background:**

Tobacco consumption peak in developed countries has passed, however, it is on the increase in many developing countries. Apart from cigarettes, consumption of local hand-rolled cigarettes such as *bidi *and *rokok daun *are prevalent in specific communities. Although factors associated with smoking initiation and cessation has been investigated elsewhere, the only available data for Malaysia is on prevalence. This study aims to investigate factors associated with smoking initiation and cessation which is imperative in designing intervention programs.

**Methods:**

Data were collected from 11,697 adults by trained recording clerks on sociodemographic characteristics, practice of other risk habit and details of smoking such as type, duration and frequency. Smoking commencement and cessation were analyzed using the Kaplan-Meier estimates and log-rank tests. Univariate and multivariate Cox proportional hazard regression models were used to calculate the hazard rate ratios.

**Results:**

Males had a much higher prevalence of the habit (61.7%) as compared to females (5.8%). Cessation was found to be most common among the Chinese and those regularly consuming alcoholic beverages. Kaplan-Meier plot shows that although males are more likely to start smoking, females are found to be less likely to stop. History of betel quid chewing and alcohol consumption significantly increase the likelihood of commencement (p < 0.0001), while cessation was least likely among Indians, current quid chewers and kretek users (p < 0.01).

**Conclusions:**

Gender, ethnicity, history of quid chewing and alcohol consumption have been found to be important factors in smoking commencement; while ethnicity, betel quid chewing and type of tobacco smoked influences cessation.

## Background

Tobacco has been classified as a Group 1 carcinogen [1]. Carcinogens found abundantly in tobacco are the polycyclic aromatic hydrocarbon benzo(α)pyrene and the tobacco specific nitrosamines. Tobacco is a widely used addictive substance with an estimated 1.3 billion smokers worldwide and a global projected tobacco-induced death at over 6 million annually [2]. The role of smoking in the development of a multitude of chronic diseases such as coronary vascular diseases (CVD) [3,4] and various neoplasm [5,6] has been substantially studied. In many of the developed countries such as US and UK, the peak of tobacco consumption have passed where prevalence of smoking has been declining over several decades; whereas in developing countries, smoking prevalence especially among men is increasing and have exceeded 50% [7].

Variations can be seen in the types of tobacco consumed across different countries. The most common type of tobacco consumption is in the form of commercial cigarettes which is widely smoked all over the world. Another form of commercially available smoked tobacco is kretek (clove cigarettes) which is commonly consumed in Indonesia [8,9]. Apart from commercially available cigarettes, other forms of tobacco consumption are local hand-rolled cigarettes such as *bidi *in India [10] and *rokok daun *in Malaysia [11]. Tobacco may also be chewed and sometimes kept in contact with the oral mucosa together with betel leaves and areca nut. Chewing this quid is practiced in South Asian populations in e.g. Taiwan and India.

In Malaysia, a small study among university students found smoking prevalence to be at 29% [12]. This figure is almost similar with smoking prevalence obtained from a nationwide survey of the Malaysian adults (23.3%) carried out by Zain et al. [13]. These data are comparable to that of the Japanese population where the national prevalence of smoking was found to be 24.2% [14]. Among Malaysians, apart from commercial cigarettes, the prevalence of *rokok daun*, a local hand-rolled cigarette where tobacco is wrapped in corn leaf is reported to be between 14-17% [11,15]. The use of this traditional type of tobacco was associated with living in rural areas, older average age, lower level of education, male gender, slightly lower consumption of cigarettes, higher social acceptability of smoking, and positive attitudes toward tobacco regulation [15]. The main reason for initiation of smoking was found to be stress and influence from friends, where smokers were also found to have a negative attitude towards tobacco control policies [12]. In line with other developing nations, smoking has been found to be on the increase among women. Prevalence of smoking among young urban Malaysian women was found to be at 16.8% where apart from peer pressure, significant association was established between female smoking and male siblings smoking status [16].

The factors associated with initiating [17,18] and quitting [19-21] smoking have been investigated. Peer pressure and delinquent behaviour are among the reasons cited for the development of this habit while future health concerns, occurrence of illness, financial stress and better quality of life were the main motivator for smoking cessation. To date, no such data are available in Malaysia. These data are needed to provide an insight into the designing of effective intervention programs. Therefore, this study aims to determine the factors associated with the commencement and cessation of smoking behaviour among Malaysians.

## Methods

### Study population and survey sampling design

This study is part of a nationwide survey to determine the prevalence of oral mucosal lesions in Malaysia, covering the Peninsula Malaysia and East Malaysia carried out in 1993-1994. The study population are adults aged 25 years old and above who has given informed consent from all the fourteen states, consisting of all the different ethnic groups. The survey only includes those living in private households, and excludes those residing in institutions such as hostels, hospitals, prisons, boarding houses and military barracks. The lowest prevalence of major lesions observed in a previous survey was used to calculate the sample size required for this survey, which was approximately 11,000-12,000. This calculation is based on significance level of 0.05 and 80% power of study.

A two-stage stratified random sampling was designed to select study participants. In the first stage, Enumeration Blocks (EB) was selected from the strata within the states. EBs are geographically contiguous areas of land with identifiable boundaries within sub districts or Local Authority areas. All EBs are formed within legally designated gazetted areas with each EB having around 80-120 households. In the second stage, a systematic sample of living quarters (LQ) with a random start was selected from within the EBs where all adults in the selected LQ were examined by the examiners. Ethical approval for this survey was obtained by the Medical Ethics Sub-committee, Research Review Committee, Ministry of Health Malaysia.

### Data collection

A structured questionnaire was used to collect data on smoking including cessation. Recording clerks were hired and trained to conduct interviews for data collection. The instrument was a check off type, which was pre-tested prior to the study. The questionnaire included demographic information, age of initiation, type, frequency, duration and cessation for smoking, alcohol consumption and betel quid chewing. This paper only focuses on smoking behaviour, while data on betel quid chewing has been published previously [22]. Smokers included those who smoke cigarettes, kretek and leaf tobacco. For analysis purposes, a current smoker is defined as a person who is currently smoking or has stopped smoking for less than 6 months, an ex smoker is a person who has stopped smoking for at least 6 months and ever smokers are those who are either current smokers or ex smokers.

### Statistical analysis

The Chi-square tests were used to compare the smoking prevalence rates and cessation rates for categories of different demographic characteristics in males, females and combined gender. The commencement and cessation of smoking behaviour in terms of time (age in years) were compared using the Kaplan-Meier estimates. To identify factors related to the commencement of smoking behaviour, all participants were included in the analysis where the event was defined as a person who started smoking behaviour. The period for the time-to-event was length of time between birth to the age of starting smoking. For the event of starting smoking, the follow-up time for censored observation is the age when interviewed during the survey. Ever smokers were included in the analysis to identify factors related to smoking cessation. An event of smoking cessation was defined as a person who had stopped smoking for at least six months. The period for the time-to-event was length of time between age of starting smoking behaviour to the age of stopped smoking. For the event of smoking cessation, the follow-up time for censored observation is the years of smoking when interviewed during the survey. The log-rank tests were used to compare the differences among the groups. The effects of gender, ethnicity, betel quid chewing and alcohol drinking on commencement and cessation of smoking behaviour were evaluated by univariate Cox proportional hazard regression models with each effect analyzed in models separately. All of the effects were also added in the same multivariate Cox proportional hazard regression models. The categories for duration, quantity or frequency of chewing and drinking were not added in multivariate models to prevent unnecessary collinearity. The proportional hazard assumptions were validated for all of the independent variables in Cox proportional hazard regression models. Analyses were conducted using the statistical software SAS V9 (SAS Institutes Inc., Cary, NC, USA).

## Results

Complete data were obtained from 11,697 respondents out of 11,707 respondents recruited. The subject ranged between 25-115 years old with a mean of 44.5 ± 13.9 years. There were slightly more female respondents (59.8%) compared to males. This multi ethnic population was made up predominantly by Malays (55.8%), followed by Chinese (23.9%), Indians (10%), Indigenous people (9.1%) and others (1.2%) which is in accordance with the population distribution of Malaysia.

Additional file 1: Table S1 shows the prevalence of ever smoking among Malaysians. Males had a much higher prevalence of the habit (61.7%) as compared to females (5.8%). In each of the variables studied, males had a higher ever smoking rate than females. Smoking was most commonly seen among those above 50 years old for both genders. Ethnic variation was registered. Among males, the Malays were found to have the highest prevalence (70.4%), followed closely by the 'others' (65.5%), while among females, the prevalence was highest among those in the 'others' group (17.1%).

In this population, the practice of multiple risk habits were seen where gender differences were observed in the practice of risk habits. Overall, 2.7% of males are found to be both a smoker and chewer (128/4698) as compared to only 1.9% seen in females (135/6999). Similarly, there are a higher percentage of concurrent drinkers and smokers among males (8.2%) compared to females (0.01%). Among male chewers, 72.7% were also smokers while among female chewers, only 20.3% were also smokers.

Additional file 2: Table S2 shows the proportion of smokers who have stopped smoking. The prevalence rate for smoking cessation is similar between the males (15.0%) and females (16.1%), so is the cessation pattern. Smoking cessation most commonly occurred among those above 50 years old, and among Chinese. The prevalence of quid chewers and alcohol consumers among those who stopped smoking were also similar between genders. Those who stopped smoking were consuming alcoholic beverages almost on a daily basis. The quit rate were similar across the different durations (in years) of smoking and also the number of cigarette smoked previously.

**Figure 1:**
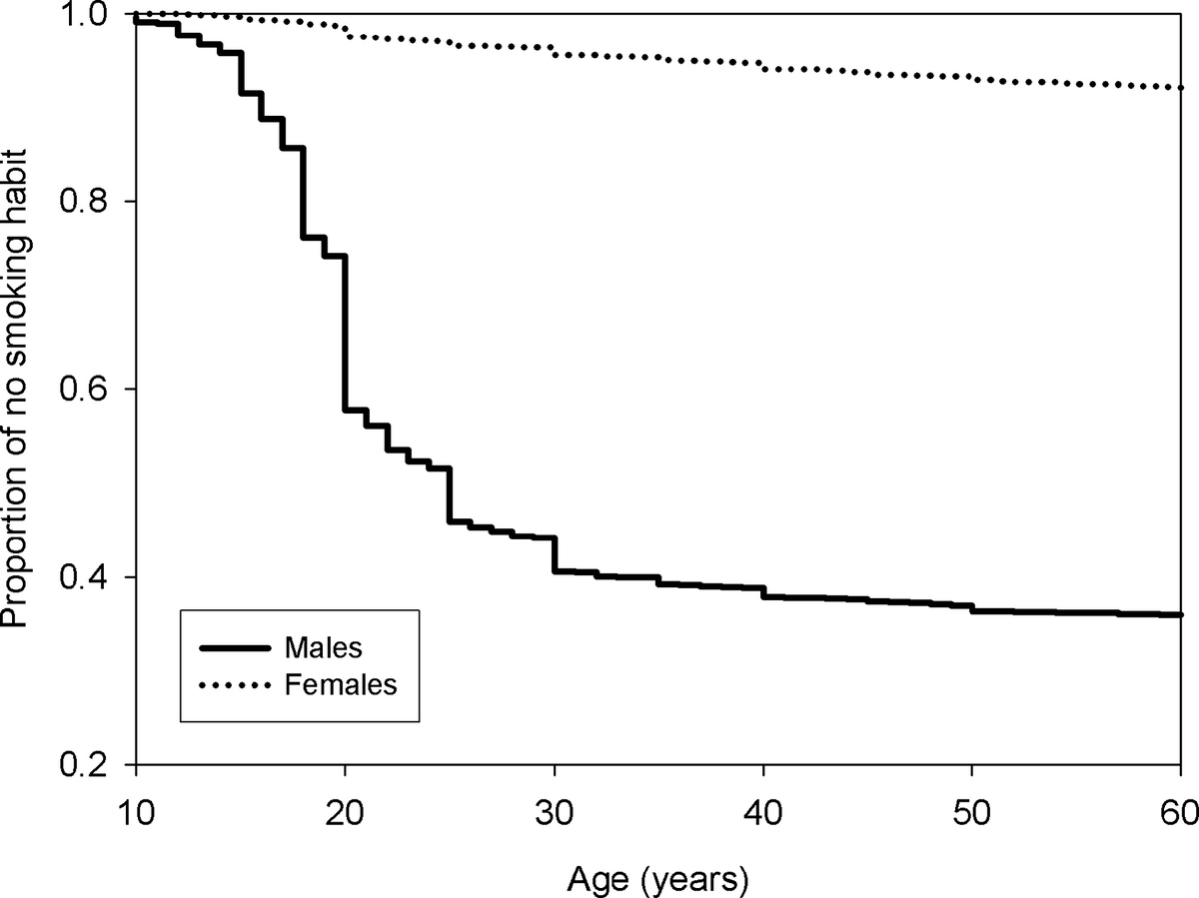
**From birth to commencement of smoking between males and females**.

**Figure 2:**
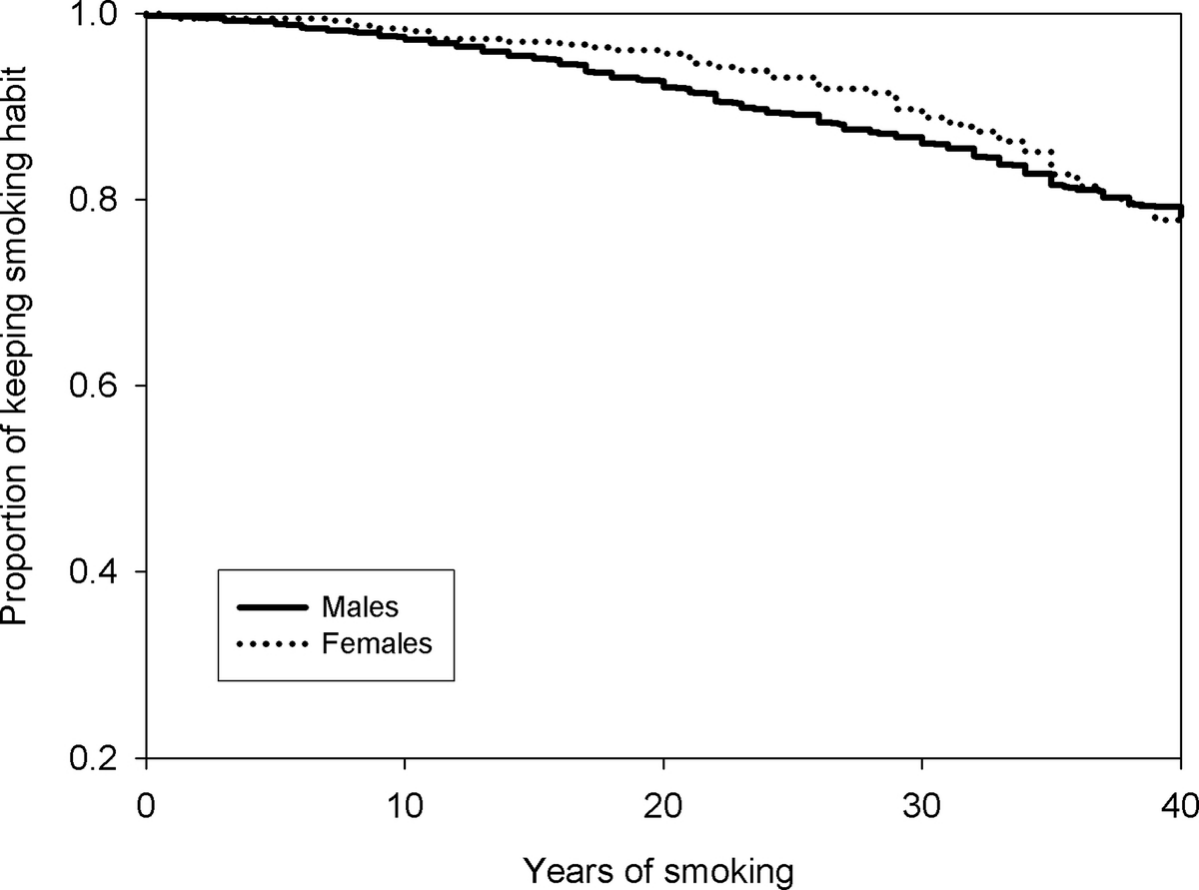
**Commencement to cessation of smoking between males and females**.

The estimated time to commencement and cessation of smoking was shown in the Kaplan-Maier plot. Figure 1 shows that the curve for developing the behaviour for females was above the curve for males, indicating that males were more likely to start smoking. Figure 2 shows that the curve for the estimated time of smoking cessation was very similar between genders. However considering that the curve for females is slightly above that for males indicates that females are more likely to keep the behaviour.

The calculation of hazard rate ratios (HRR) using Cox regression analysis were estimated to determine the factors involved in the development and cessation of smoking. With regards to developing smoking behaviour, almost all of the factors investigated showed statistically significant results (Additional file 3: Table S3). Females (HRR = 0.06, 95%CI 0.06-0.07) were significantly less likely to start smoking. In terms of ethnicity, Indians, Chinese and Indigenous people were less likely to develop the behaviour compared to Malays. Current (HRR = 2.02, 95%CI 1.77, 2.30) and past (HRR = 2.7, 95%CI 2.14, 3.40) history of quid chewing was found to significantly increase the likelihood of developing a smoking behaviour. Current alcohol drinkers were also found to be two times more likely to start smoking (HRR = 2.01, 95%CI 1.79, 2.25).

The analysis of factors associated with cessation of smoking is shown in Additional file 4: Table S4. Smoking cessation was least likely to be found among Indians (HRR = 0.40, 95%CI 0.25-0.64) and current quid chewers (HRR = 0.60, 95%CI 0.40, 0.90). Among the different types of tobacco smoked, kretek users were least likely to quit smoking (HRR = 0.10, 95%CI 0.06, 0.19).

## Discussions

In Malaysia, smoking is more prevalent among males. This trend is in agreement with other studies on various populations worldwide [23,24]. Furthermore, ethnic variation in the prevalence of this behaviour was observed which is also seen in other populations [25]. Apart from smoking, other risk habits for oral mucosal lesions such as alcohol consumption and betel quid chewing is also prevalent. The practice of multiple habits simultaneously in this study which is more commonly found among males is in line with other studies [26].

Data on smoking behaviour from other studies are mainly on only one ethnic group for the country, for example data on Indians from India, and on the Chinese from China and Taiwan. However, there is a need to understand the smoking behaviours among the different ethnic groups. As Malaysia is one of the few countries in Asia that has various races living in the same environment, this study provided a good platform for comparing smoking behaviour among different ethnicity. The Malays were found to be significantly more likely to start smoking, while smoking cessation was least likely among the Indians. It was also observed that the proportion of quitting smoking was highest among the Chinese, however multivariate analysis did not find any statistical significance. The ethnic variation seen could be attributed to their attitude towards health. In a national study on 16,127 Malaysians, the Malays and Indians were found to have the highest prevalence of obesity while it was lowest among the Chinese [27], which probably reflects their level of awareness, knowledge and interest on health and nutrition issues. It was also reported that the Chinese scored the highest for general knowledge on breast cancer, followed by the Malays and Indians [28]. Furthermore, Malay and Indian women were also reported to be the more likely to present with late stage breast cancer as compared to the Chinese [29], which indicates that generally, the Chinese are more concerned of their health status.

History of betel quid chewing has been significantly found to increase the likelihood of smoking commencement. This could be explained by the fact that tobacco included in the quid would probably already promote nicotine dependency, thus leading to the initiation of smoking. Concurrently, this study also found that those currently practicing the habit of betel quid chewing are less likely to stop smoking. An interesting observation is that among males, univariate analysis found that those who quit smoking were more likely to be regular alcohol consumers, which is not in agreement with other studies [26,30]. This higher rate of smoking cessation among alcohol consumers in this population could be due to the fact that the practice of another habit that promotes addiction such as consuming alcohol eases and lessen the withdrawal symptoms in the process of removing the addiction and dependency to nicotine that is found in cigarettes. Among the different type of tobacco use, kretek users were found to be the least likely individuals to stop smoking. This could be attributed to the higher nicotine content in kretek as compared to cigarettes [31], which promotes greater dependency among its users.

Interestingly, in this study, univariate analysis found no dose-response relationship as found by other researchers [32,33], where in this present study, no difference was seen in the quit rate across the years of smoking and number of cigarettes smoked previously. This finding could be explained by the fact that most of those quitting smoking are of the Chinese ethnicity, which has been shown to be more health conscious compared to the other ethnic groups. Researchers have suggested that the main reason for quitting smoking is health concerns [19,21]. Thus, awareness and willingness to quit smoking based on health reasons supersede the number of years and sticks smoked previously. On the other hand, as there is currently a declining trend of social acceptability of tobacco use, there is also the risk of underreporting by the respondents on their smoking status [34].

In line with findings by other researchers, males were found to be more likely to start smoking earlier. This could be largely attributed to the sociocultural environment of the population. Males are more likely to initiate the behaviour due to peer pressure and as a sign of masculinity and machoism [17,18,35,36], while females are less likely to start smoking as it is always associated with social stigma. In Asian countries such as Malaysia, it is perceived to be alright if males smoke. However, it is considered to be culturally inappropriate if a female smokes indicating that they are perceived as ill-mannered and 'bad' [35-37]. Other reasons cited by females for not smoking are health concerns, family values and spouse influence [38]. Although smoking is generally more prevalent among males, literature has showed that in recent years, there is a trend of an increasing prevalence among females [39]. Of interest to note, this study found that the female smokers are less likely to quit smoking as compared to males which is in concordance with other studies [40,41]. A plausible explanation relates to the main concern that women have when thinking to quit which is weight gain. It was reported that the barriers in smoking cessation among fifty percent of female smokers are concerns about weight gain [42]. Their fears are not unfounded as studies have shown that females gain more weight than males after quitting smoking [43]. This concern may be reflected in our study population where none of the female smokers in the 20-25 age group were found to quit smoking.

Although cigarette smoking has been accepted worldwide as a risk factor for an array of chronic, potentially fatal diseases such as cancer, there are still pockets of society that could not see the link between tobacco and disease [21]. This present study found that smoking is still very prevalent, especially among the males in Malaysia. This scenario could be due to the lack of emphasis on consequences of practicing this behaviour over an extended period of time prior to the conduct of the study. Presently, the government has introduced various campaigns and health policies targeted at reinforcing the adverse effect of this behaviour, not just on health, but also the social and economic impact on the individual and society such as the nationwide 'Say No' campaign. Policies targeting smokers were introduced where graphic warnings on the effect of smoking on lungs, oral cavity and babies were displayed on cigarette boxes. Strategies to reduce the prevalence of smoking were also carried out through the annual increase in the tax on cigarettes.

In relation to the ethnic variation in the smoking behaviour observed in this population, efforts on future policies and strategies by health personnel needs to be targeted and tailored towards the high risk groups as identified in this study. The reasons that influence the initiation and cessation of smoking should also be further investigated so that health authorities are better equipped to formulate future cessation programs more efficiently. Furthermore, future efforts need to also target and emphasize the hazards of other high risk behaviours, such as concurrently smoking, chewing betel quid and drinking which is currently seen in this population.

## Conclusions

This study found that smoking in Malaysia is most common among Malay males. Factors that influence the development of this behaviour are gender, ethnicity, history of quid chewing and alcohol consumption; while ethnicity, practice of quid chewing and type of tobacco smoked are important factors for cessation.

## Competing interests

The authors declare that they have no competing interests.

## Authors' contributions

WMNG have made substantial contribution in the conception of manuscript framework, interpretation of data analysis and has drafted the manuscript. IAR helped conceived of the study, participated in the study design conception and coordination and helped to critically revise the manuscript. YHY performed the statistical analysis, interpretation of data and helped to critically revise the manuscript. NAT conceived and participated in the study design conception and project coordination. NA help in study design conception and revision of the manuscript and NI helped in acquisition of funding, involved in study design conception and coordination and revised the manuscript. PCG is involved in study design conception and coordination, provided substantial contribution to sampling estimation and revised the manuscript. TA and YH helped conceived and participated in the study design conception, and revised the manuscript. RBZ conceived the study design, led and coordinate the study/research group, ensure quality control of data and critically revise the manuscript. All authors read and approved the final manuscript.

## Pre-publication history

The pre-publication history for this paper can be accessed here:

http://www.biomedcentral.com/1471-2458/12/207/prepub

## Supplementary Material

Additional file 1 **Table S1 Smoking habit prevalence figures of males and females distributed according to different demographic characteristics**. Table 1 tabulated the practice of smoking across different sociodemographic characteristics of the study population such as age, ethnicity, betel quid chewing and drinking habit. Click here for file

Additional file 2 **Table S2 Proportion of smokers who stopped smoking with different demographic characteristics distributed according to males and females**. Table 2 tabulated the cessation of smoking across different sociodemographic characteristics such as age, ethnicity, betel quid chewing habit, drinking habit and duration/frequency of smoking. Click here for file

Additional file 3 **Table S3 Univariate and multivariate analysis of smoking habit from birth until commencement of smoking**. Table 3 shows the results of univariate and multivariate analysis of the association between selected variables and commencement of smoking. Click here for file

Additional file 4 **Table S4 Univariate and multivariate analysis of smoking habit from inception until cessation**. Table 4 shows the results of univariate and multivariate analysis of the association between selected variables and cessation of smoking. Click here for file
