# Supplementary material for: Factors affecting commencement and cessation of smoking behaviour in Malaysian adults
Source: BMC Public Health. 2012 Mar 19;12:207. doi: 10.1186/1471-2458-12-207 (PMC3349505; doi:10.1186/1471-2458-12-207)
Supplement: Additional file 1 — Table S1 Smoking habit prevalence figures of males and females distributed according to different demographic characteristics. Table 1 tabulated the practice of smoking across different sociodemographic characteristics of the study population such as age, ethnicity, betel quid chewing and drinking habit. [file 1471-2458-12-207-S1.PDF]

Table 1. Ever smoking prevalence figures of males and females distributed according to different demographic characteristics

Variables	Item	Males (n=4698)			Females (n=6999)			Total		
		Total no of subjects	No of ever smokers, n (%)	p value	Total no of subjects	No of ever smokers, n (%)	p value	Total no of subjects	No of ever smokers, n (%)	p value
Total		4698	2898 (61.7)		6999	409 (5.8)		11697	28.3	
Age group	25-30	676	382 (56.5)	<.001	1297	20 (1.5)	<.001	1973	20.4	<.0001
	31-40	1237	708 (57.2)		2270	34 (1.5)		3507	21.2	
	41-50	1044	626 (60.0)		1522	65 (4.3)		2566	26.9	
	51+	1741	1182 (67.9)		1910	290 (15.2)		3651	40.3	
Ethnicity	Malays	2605	1833 (70.4)	<.001	3924	219 (5.6)	<.001	6529	31.4	<.0001
	Indigenous people	432	267 (61.8)		580	81 (14.0)		1012	34.4	
	Chinese	1097	517 (47.1)		1701	85 (5.0)		2798	21.5	
	Indians	477	244 (47.0)		689	6 (0.9)		1166	19.7	
	Others*	87	57 (65.1)		105	16 (19.8)		192	39.1	
Chewer	Yes	176	128 (72.7)	<0.01	666	135 (20.3)	<.001	842	31.2	0.0475
	No	4522	2770 (61.3)		6333	274 (4.3)		10855	28.0	
Duration (years)	0-19	72	63 (87.5)	<0.05	81	10 (12.3)	<0.05	153	47.7	<.0001
	20-29	48	37 (77.1)		24	3 (12.5)		72	55.6	
	30+	106	75 (70.8)		168	9 (5.4)		274	30.7	

Variables	Item	Males (n=4698)			Females (n=6999)			Total		
		Total no of subjects	No of ever smokers, n (%)	p value	Total no of subjects	No of ever smokers, n (%)	p value	Total no of subjects	No of ever smokers, n (%)	p value
No of quid chewed	0-4	160	131 (81.9)	<0.05	3	0 (0.0)	0.5704	163	80.4	0.0258
	5-9	35	25 (71.4)		2	0 (0.0)		37	67.6	
	10+	31	19 (61.3)		2	1 (50.0)		33	60.6	
Type of quid chewed	Areca + tobacco	50	19 (38.0)	<.001	280	23 (8.2)	<.001	330	12.7	
	Areca only	149	134 (89.9)		410	128 (31.2)		559	46.9	
	Tobacco only	6	2 (33.3)		12	0 (0.0)		18	11.1	
	No areca + tobacco	21	20 (95.2)		35	12 (34.3)		56	57.1	
Alcohol drinker	Yes	517	383 (74.1)	<.001	3	1 (33.3)	<.001	520	73.8	<.0001
	No	4181	2515 (60.2)		164	59 (36.0)		4345	59.2	
Duration (years)	0-10	141	96 (68.1)	<0.01	20	1 (5.0)	<0.01	161	60.2	<.0001
	11-25	209	149 (71.3)		25	8 (32.0)		234	67.1	
	26+	167	138 (82.6)		1	0 (0.0)		168	82.1	
Frequency of drinking/ week	<1 time	257	180 (70.0)	<0.01	4	1 (25.0)	0.6837	261	69.3	<.0001
	1-2 times	110	77 (70.0)		192	57 (29.7)		302	44.4	
	3-5 times	51	45 (88.2)		1	1 (100.0)		52	88.5	
	Almost daily	99	81 (81.8)		1	0 (0.0)		100	81.0	

Variables	Item	Males (n=4698)			Females (n=6999)			Total		
		Total no of subjects	No of ever smokers, n (%)	p value	Total no of subjects	No of ever smokers, n (%)	p value	Total no of subjects	No of ever smokers, n (%)	p value
Cigarette	Yes	1994	1994 (100.0)	<.001	162	162 (100.0)	<.001	2156	99.9	<.0001
	No	2704	904 (33.4)		6837	247 (3.6)		9541	12.1	
Kretek	Yes	396	396 (100.0)	<.001	14	14 (100.0)	<.001	410	100.0	<.0001
	No	4302	2502 (58.2)		6985	395 (5.7)		11287	25.7	
Leaf tobacco	Yes	602	602 (100.0)	<.001	188	188 (100.0)	<.001	790	99.6	<.0001
	No	4096	2296 (56.1)		6811	221 (3.2)		10907	23.1	

Numbers do not add up due to missing value
Smokers includes those who smoke cigarettes, kretek and leaf tobacco
*Others: All other ethnic groups that does not fall into the stated categories, ie Orang Asli, etc
