# Supplementary material for: Factors affecting commencement and cessation of smoking behaviour in Malaysian adults
Source: BMC Public Health. 2012 Mar 19;12:207. doi: 10.1186/1471-2458-12-207 (PMC3349505; doi:10.1186/1471-2458-12-207)
Supplement: Additional file 2 — Table S2 Proportion of smokers who stopped smoking with different demographic characteristics distributed according to males and females. Table 2 tabulated the cessation of smoking across different sociodemographic characteristics such as age, ethnicity, betel quid chewing habit, drinking habit and duration/frequency of smoking. [file 1471-2458-12-207-S2.PDF]

Table 2. Proportion of ever smokers who stopped smoking with different demographic characteristics distributed according to males and females

Variables	Item	Males (n=2898)			Females (n=409)			Total		
		Total no of ever smokers	No of stopped smoking, n (%)	p value	Total no of ever smokers	No of stopped smoking, n (%)	p value	Total no of ever smokers	No of stopped smoking, n (%)	p value
Total		2898	434 (15.0)		409	66 (16.1)		3307	15.1	
Age group	25-30	382	24 (6.3)	<.001	20	0 (0.0)	<0.05	402	6.0	<.0001
	31-40	708	77 (10.9)		34	6 (17.6)		742	11.2	
	41-50	626	91 (14.5)		65	4 (6.2)		691	13.7	
	51+	1182	242 (20.5)		290	56 (19.3)		1472	20.2	
Ethnicity	Malays	1833	277 (15.1)	<0.01	219	35 (16.0)	0.5954	2052	15.2	0.0008
	Indigenous people	267	31 (11.6)		81	11 (13.6)		348	12.1	
	Chinese	517	99 (19.1)		85	18 (21.2)		602	19.4	
	Indians	224	23 (10.3)		6	0 (0.0)		230	10.0	
	Others*	41	2 (4.9)		16	2 (12.5)		75	8.0	
Chewer	Yes	175	33 (18.9)	0.1377	163	30 (18.4)	0.3101	338	18.6	0.0566
	No	2723	410 (14.7)		246	36 (14.6)		2969	14.7	
Duration (years)	0-19	63	8 (12.7)	0.2746	45	9 (20.0)	0.8855	108	15.7	0.6426
	20-29	37	9 (24.3)		32	5 (15.6)		68	20.3	
	30+	75	16 (21.3)		86	16 (18.6)		161	19.9	

Variables	Item	Males (n=2898)			Females (n=409)			Total		
		Total no of ever smokers	No of stopped smoking, n (%)	p value	Total no of ever smokers	No of stopped smoking, n (%)	p value	Total no of ever smokers	No of stopped smoking, n (%)	p value
No of quid chewed	0-4	131	27 (20.6)	0.5330	109	24 (22.0)	0.2324	240	21.3	0.1391
	5-9	25	4 (16.0)		33	4 (12.1)		58	13.8	
	10+	19	2 (10.5)		21	2 (9.5)		40	10.0	
Type of quid	Areca + tobacco	19	3 (15.8)	0.0737	23	3 (13.0)	0.0867	42	14.3	0.0082
	Areca only	134	22 (16.4)		128	22 (17.2)		262	16.8	
	Tobacco only	2	0 (0.0)		0	-		2	0.0	
	No areca & tobacco	20	8 (40.0)		12	5 (41.7)		32	40.6	
Alcohol drinker	Yes	383	60 (15.7)	0.6846	23	3 (13.0)	0.6781	406	15.5	0.8112
	No	2515	374 (14.9)		386	63 (16.3)		2901	15.1	
Duration (years)	0-10	96	11 (11.5)	0.4196	6	0 (0.0)	0.2057	102	10.8	0.2853
	11-25	149	25 (16.8)		5	0 (0.0)		154	16.2	
	26+	138	24 (17.4)		12	3 (25.0)		150	18.0	
Frequency of drinking/ week	<1 time	180	34 (18.9)	<0.05	15	2 (13.3)	0.3985	195	18.5	0.0189
	1-2 times	77	4 (5.2)		5	0 (0.0)		82	4.9	
	3-5 times	45	6 (13.3)		0	-		45	13.3	
	Almost daily	81	16 (19.8)		3	1 (33.3)		84	20.2	

Variables	Item	Males (n=2898)			Females (n=409)			Total		
		Total no of ever smokers	No of stopped smoking, n (%)	p value	Total no of ever smokers	No of stopped smoking, n (%)	p value	Total no of ever smokers	No of stopped smoking, n (%)	p value
Years of smoking	0-19	1165	179 (15.4)	0.8703	125	16 (12.8)	0.4462	1290	15.1	0.9899
	20-29	663	96 (14.5)		85	16 (18.8)		748	15.0	
	30+	1070	159 (14.9)		199	34 (17.1)		1269	15.2	
Cigarette	Yes	1994	169 (8.5)	<.001	162	10 (6.2)	<.001	2156	8.3	<.0001
	No	904	265 (29.3)		247	56 (22.7)		1151	27.9	
No of cigarette	0-9	459	39 (8.5)	0.8548	70	4 (5.7)	0.3991	529	8.1	0.6811
	10-19	709	57 (8.0)		54	2 (3.7)		763	7.7	
	20+	826	73 (8.8)		38	4 (10.5)		864	8.9	
Kretek	Yes	396	11 (2.8)	<.001	14	0 (0.0)	0.0949	410	2.7	<.0001
	No	2502	423 (16.9)		395	66 (16.7)		2897	16.9	
No of kretek	0-5	128	6 (4.7)	0.1126	7	0 (0.0)		135	4.4	0.1210
	6-10	118	4 (3.4)		3	0 (0.0)		121	3.3	
	11+	150	1 (0.7)		4	0 (0.0)		154	0.6	
Leaf tobacco	Yes	602	43 (7.1)	<.001	188	14 (7.4)	<.001	790	7.2	<.0001
	No	2296	391 (17.0)		221	52 (23.5)		2517	17.6	
No of leaf tobacco	0-5	149	12 (8.1)	0.3064	110	9 (8.2)	0.3182	259	8.1	0.5724
	6-10	234	20 (8.5)		55	2 (3.6)		289	7.6	
	11+	219	11 (5.0)		23	3 (13.0)		242	5.8	

Numbers do not add up due to missing value
Smokers includes those who smoke cigarettes, kretek and leaf tobacco
*Others: All other ethnic groups that does not fall into the stated categories, ie Orang Asli, etc
