# Supplementary material for: Factors affecting commencement and cessation of smoking behaviour in Malaysian adults
Source: BMC Public Health. 2012 Mar 19;12:207. doi: 10.1186/1471-2458-12-207 (PMC3349505; doi:10.1186/1471-2458-12-207)
Supplement: Additional file 3 — Table S3 Univariate and multivariate analysis of smoking habit from birth until commencement of smoking. Table 3 shows the results of univariate and multivariate analysis of the association between selected variables and commencement of smoking. [file 1471-2458-12-207-S3.PDF]

Table 3. Univariate and multivariate analysis of smoking habit from birth until commencement of smoking

Variables	Item	Univariate			Multivariate		
		Hazard rate ratio	95%CI	p value	Hazard rate ratio	95%CI	p value
Gender	Male	1.00†			1.00†		
	Female	0.07	(0.06, 0.07)	<0.001	0.06	(0.06, 0.07)	<0.001
Ethnicity	Malay	1.00†			1.00†		
	Indigenous people	1.14	(1.02, 1.28)	<0.05	0.81	(0.71, 0.91)	<0.001
	Chinese	0.63	(0.58, 0.69)	<0.001	0.53	(0.48, 0.58)	<0.001
	Indian	0.59	(0.52, 0.68)	<0.001	0.41	(0.36, 0.47)	<0.001
	Others*	1.31	(1.00, 1.70)	<0.05	0.98	(0.74, 1.25)	0.7597
Betel quid chewer	No	1.00†			1.00†		
	Ex	2.53	(2.02, 3.19)	<0.001	2.70	(2.14, 3.40)	<0.001
	Current	1.06	(0.94, 1.21)	0.3462	2.02	(1.77, 2.30)	<0.001
Alcohol drinker	No	1.00†			1.00†		
	Current	3.22	(2.90, 3.57)	<0.001	2.01	(1.79, 2.25)	<0.001

† Reference category

*Others: All other ethnic groups that does not fall into the stated categories, ie Orang Asli, etc
