# Supplementary material for: Factors affecting commencement and cessation of smoking behaviour in Malaysian adults
Source: BMC Public Health. 2012 Mar 19;12:207. doi: 10.1186/1471-2458-12-207 (PMC3349505; doi:10.1186/1471-2458-12-207)
Supplement: Additional file 4 — Table S4 Univariate and multivariate analysis of smoking habit from inception until cessation. Table 4 shows the results of univariate and multivariate analysis of the association between selected variables and cessation of smoking. [file 1471-2458-12-207-S4.PDF]

Table 4. Univariate and multivariate analysis of smoking habit from inception until cessation

Variables	Item	Univariate			Multivariate		
		Hazard rate ratio	95%CI	p value	Hazard rate ratio	95%CI	p value
Gender	Male	1.00†			1.00†		
	Female	0.95	(0.73, 1.23)	0.6877	0.79	(0.60, 1.05)	0.1105
Ethnicity	Malay	1.00†			1.00†		
	Indigenous people	0.87	(0.63, 1.21)	0.4059	0.68	(0.48, 0.95)	<0.05
	Chinese	1.21	(0.98, 1.50)	0.0819	0.87	(0.69, 1.09)	0.2300
	Indian	0.66	(0.43, 1.00)	0.0511	0.40	(0.25, 0.64)	<0.001
	Others*	0.64	(0.28, 1.43)	0.2467	0.66	(0.29,1.48)	0.2141
Betel quid chewer	No	1.00†			1.00†		
	Ex	2.18	(1.54, 3.10)	<0.001	1.55	(1.08, 2.23)	<0.05
	Current	0.55	(0.38, 0.80)	<0.01	0.60	(0.40, 0.90)	<0.05
Alcohol drinker	No	1.00†			1.00†		
	Current	1.01	(0.77, 1.31)	0.9532	1.13	(0.83, 1.52)	0.4348
Smoker	No	1.00†			1.00†		
	Cigarette user	0.41	(0.34, 0.49)	<0.001	0.21	(0.17, 0.25)	<0.001
	Kretek user	0.23	(0.12, 0.41)	<0.001	0.10	(0.06, 0.19)	<0.001
	Leaf tobacco user	0.26	(0.20, 0.34)	<0.001	0.13	(0.10, 0.17)	<0.001

† Reference category

*Others: All other ethnic groups that does not fall into the stated categories, ie Orang Asli, etc
